# Polymorphisms in ERCC1, GSTs, TS and MTHFR predict clinical outcomes of gastric cancer patients treated with platinum/5-Fu-based chemotherapy: a systematic review

**DOI:** 10.1186/1471-230X-12-137

**Published:** 2012-09-29

**Authors:** Zhen Wang, Jun-qiang Chen, Jin-lu Liu, Xin-gan Qin, Yuan Huang

**Affiliations:** 1 Department of Gastrointestinal Surgery, The First Affiliated Hospital of Guangxi Medical University, 6 Shuangyong Road, Nanning, Guangxi Zhuang Autonomous Region, 530021, China

**Keywords:** Gastric cancer, Genetic polymorphism, Chemotherapy, Meta-analysis

## Abstract

**Background:**

Despite genetic polymorphism in response to platinum/5-Fu chemotherapy in gastric cancer (GC) has been studied, data reported so far are conflicting and critical consideration is needed before translation to the treatment of GC.

**Methods:**

We performed a meta-analysis by using 20 eligible studies to examine polymorphisms of ERCC1, GSTs, TS and MTHFR in predicting clinical outcomes (response rate, overall survival and toxicity) of GC patients treated with platinum/5-Fu-based chemotherapy. The association was measured using random/fixed effect odds ratios (ORs) or hazard ratios (HRs) combined with their 95% confidence intervals (CIs) according to the studies’ heterogeneity. Statistical analysis was performed with the software STATA 9.0 package.

**Results:**

No significant association was found between response rate and genetic polymorphism in TS, MTHFR, ERCC1, GSTM1 and GSTP1. However, response rate was higher in GSTT1 (+) genotype compared with GSTT1 (−) genotype (T-/T+: OR=0.67, 95% CI: 0.47–0.97). With regard to long term outcomes, we could observe a significant longer overall survival in TS 3R/3R [(2R2R+2R3R)/3R3R: HR=1.29, 95% CI: 1.02–1.64] and GSTP1 GG/GA [(GG+AG)/AA: HR=0.51, 95% CI: (0.39, 0.67)] genotypes. In addition, significant association was demonstrated between toxicity and genetic polymorphism in TS, MTHFR and GSTP1 in included studies.

**Conclusion:**

Polymorphisms of ERCC1, GSTs, TS and MTHFR were closely associated with clinical outcomes of GC patients treated with platinum/5-Fu-based chemotherapy. Studies with large sample size using the method of multi-variant analyses may help us to give more persuasive data on the putative association in future.

## Background

In worldwide, gastric cancer (GC) remains one of the major causes of cancer-related death worldwide [1]. Surgery is the primary modality for managing early-stage and locally-advanced disease. However, even after gastrectomy, many patients relapse with local recurrence or distant metastasis [2]. In addition, approximately 20–30% of patients have inoperable disease at diagnosis. Therefore, the majority of patients need a systemic therapy at some point in their disease.

Palliative chemotherapy for advanced GC has been widely accepted as a standard treatment for several decades. And recent studies have demonstrated that peri-operative adjuvant chemotherapy (pre- or post-operative) can improve survival and quality of life in patients with GC [3]; however, expected survival for the advanced disease is generally poor (less than 1 year). Until now, 5-fluorouracil (5-Fu) and platinum are the most common drugs used for GC both in adjuvant and advanced settings, although there are no standard combination regimens [4]. Additionally, efficacy outcomes for a number of new agents (such as paclitaxel, oxaliplatin and capecitabine) have not shown definitive clinical benefit or superiority to older drugs in patients with advanced GC [5-7]; and in some patients therapy results in severe, unpredictable toxicity without any tumor response. Consequently, in order to allow the discernment of patients in whom a particular therapy will exert a real benefit, it is crucial to identify factors relevant to response to fluorouracil/platinum as well as factors predisposed to the development of severe toxicity. In this regard, pharmacogenetics, a research field identifying inherited genetic variability which may affect treatment outcomes, could allow a tailored management regimen that maximizes clinical response while limiting the adverse effects of treatment [8,9].

A growing body of evidence suggests that inter-individual variation in drug-metabolizing enzymes and nucleotide excision repair (NER) system may affect anticancer drug efficacy by influencing DNA repair or related enzyme activities [10]. Recently, many studies finds that genes involved in DNA detoxification (glutathione S-transferases, GSTs) and repair (excision repair cross complementing 1, ERCC1) control the effects of platinum [11,12], while methylene tetrahydrofolate reductase (MTHFR) and thymidylate synthase (TS) are associated with 5-Fu metabolism [11,13]. Despite genetic polymorphism in response to platinum/5-Fu chemotherapy in GC has been reported [14], data reported so far are conflicting and critical consideration is needed before translation to the treatment of GC. Therefore, a systematic review is solely needed to provide a comprehensive and up-to-date overview concerning possible roles for genetic polymorphisms in GC treatment.

In this study, we assessed literatures existing and conducted a meta-analysis to examine polymorphisms of ERCC1, GSTs, TS and MTHFR in predicting clinical outcomes of GC patients treated with platinum/5-Fu-based chemotherapy.

## Methods

### Search strategy

A computer-aided search of the Pubmed/Medline and Embase was performed to identify relevant and available published articles by using the following search phrases: gastric cancer/carcinoma/tumor/tumour/neoplasm, stomach cancer/carcinoma/tumor/tumour/neoplasm, polymorphism/polymorphisms and chemotherapy. The upper limit of search date was not limited, and the lower limit was January 2012. Both free text and MeSH search for keywords were employed. The language that the papers were written in was not restricted. To identify more potentially relevant studies, reference lists from selected studies through electronic searching were hand searched.

### Inclusion and exclusion criteria

The inclusion criteria of this meta-analysis were as follows: 1) pathologically confirmed GC with a measurable lesion; 2) no concurrent uncontrolled medical illness; 3) patients receiving no other adjuvant treatment, such as radiotherapy or immunotherapy; 4) clinical outcomes [response rate (RR), overall survival (OS) or toxicity) about genetic polymorphisms [ERCC1-118, GSTs (GSTM1, GSTP1-105 or GSTT1), TS 5’-untranslated region or MTHFR-667] in GC patients treated with platinum/5-Fu-based chemotherapy were reported.

The exclusion criteria were: 1) included patients with carcinoma other than the stomach; 2) in vitro studies; 3) studies were not original research, such as review article; 4) platinum/5-Fu were not included in the chemotherapeutic regimens. We included literatures with largest sample size for repetitive publications.

### Data extraction

Two authors (Zhen Wang and Jun-qiang Chen) extracted data independently from all eligible studies using predefined tables, which included items as follows: first author, publication time, country and ethnicity of the patients, molecular marker, sample size, evaluation criteria, chemotherapeutic regimens and clinical outcomes (RR, OS and toxicity). If necessary, the authors of the original literatures were contacted for available data. Disagreements were resolved by consensus.

### Statistical analysis

Hazard ratios (HRs) and their 95% confidence intervals (CIs) for OS were obtained from each primary study. In case the data were not directly recorded in primary reports, we calculated HR and their 95% CIs from the survival curves using published methodology [15,16]. Kaplan–Meier curves of included studies were read by Engauge Digitizer version 2.11 (free software downloaded from http://sourceforge.net). HR calculation spreadsheet was freely downloaded from http://www.trialsjournal.com/content/supplementary/1745-6215-8-16-s1.xls. The odds ratio (OR) for RR and HRs for OS were calculated based on a fixed-effect model first by using STATA 9.0 package. Heterogeneity between included studies was tested using χ^2^ test (considered significant if *P*<0.10). If heterogeneities were present, one of the following measures was used to attempt to explain them: (1) subgroup analysis; (2) sensitivity analysis; or (3) random-effect model for meta-analysis. All *P* values were two-sided and all CIs had a two-sided probability coverage of 95%.

## Results

### Study selection and description

**Figure 1:**
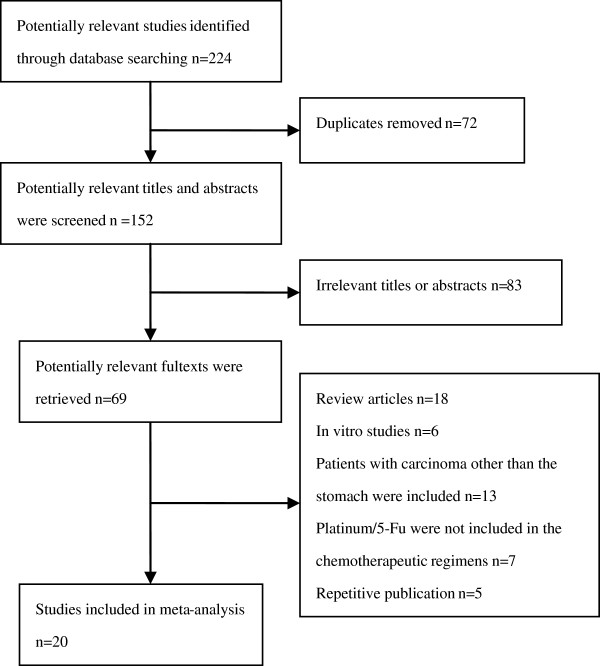
QUORUM flow chart for studies.

According to the search strategy referred, a total of 224 literatures were yielded: 130 in PubMed and 94 in EMBASE. By browsing the titles and abstracts, we found that lots of articles were irrelevant and some were identified duplicately, thus 69 articles remained for potential inclusion and were obtained in full-text version. After reviewing the full text, 49 literatures were excluded. The main reasons for excluding studies were as follows: study type (review articles and in vitro studies), participants (inclusion of patients with carcinoma other than the stomach), interventions (exclusion of platinum/5-Fu in the chemotherapeutic regimens) and repetitive publication. Finally 20 studies (2189 patients) were considered eligible for inclusion [11,14,17-34]. The process of study selection was listed in Figure 1.

Among the 20 included studies, the number of TS, MTHFR, ERCC1 and GSTs polymorphism study was 10 (952 patients), 9 (988 patients), 10 (1080 patients) and 10 (1187 patients), respectively. The sample size varied from 25 to 200 and the publication time was from 2002 to 2011. Participants were Asian and European. The main characteristics of the 20 included studies were listed in Table 1.

**Table 1:** **Characteristics of studies includedin the systematic review**

**Study (reference)**	**Patients**	**Molecular marker**	**Ethnicity (Country)**	**Evaluation criteria**	**Sample size**	**Chemotherapeutic regimens**	**Outcomes**
Goekkurt 2006, [11]	Patients with advanced GC	TS, GSTs, MTHFR, ERCC1-118	European (Germany)	RECIST	52	5-Fu+cisplatin + leucovorin	RR and OS
Ruzzo 2006 [14]	Patients with advanced GC	TS, GSTs, MTHFR, ERCC1-118	European (Italy)	Others	175	fluorouracil/cisplatin	RR and OS
Shim 2010 [17]	Patients with recurrent or metastatic GC	GSTs	Asian (Korea)	RECIST	200	Paclitaxel/docetaxel +cisplatin	RR and OS
Park 2011 [18]	Patients with metastatic GC	ERCC1-118	Asian (Korea)	RECIST	108	S-1 + cisplatin	RR and OS
Han 2010 [19]	Patients with recurrent or metastatic GC	TS, ERCC1-118	Asian (Korea)	RECIST	38	5-Fu+leucovorin +oxaliplatin	RR and OS
Stocker 2009 [20]	Neoadjuvant chemotherapy for locally advanced GC without distant metastasis	ERCC1-118	European (Germany)	Others	178	5-Fu+leucovorin +cisplatin	RR and OS
Seo 2009 [21]	Patients with recurrent or metastatic GC	TS, GSTs, ERCC1-118	Asian (Korea)	RECIST	94	5-Fu+ oxaliplatin/irinotecan	RR, OS and toxicity
Liu 2011 [22]	Patients with advanced GC	GSTs, ERCC1-118	Asian (China)	NR	126	5-FU+leucovorin +oxaliplatin	OS
Ott 2008 [23]	Neoadjuvant chemotherapy for locally advanced GC without distant metastasis	GSTs	European (Germany)	Others	139	5-Fu+leucovorin + cisplatin	RR and OS
Li 2010 [24]	Patients with advanced GC	GSTs	Asian (China)	Others	92	5-Fu +oxaliplatin	RR, OS and toxicity
Lu 2004 [25]	Patients with advanced GC	MTHFR	Asian (China)	WHO	75	5-Fu+leucovorin	RR and toxicity
Huang 2009 [26]	Patients with GC after curative surgery	GSTs, ERCC1-118	Asian (China)	NR	102	5-Fu+leucovorin +oxaliplatin	OS
Shitara 2010 [27]	Patients with inoperable GC	TS, MTHFR	Asian (Japan)	NR	132	5-Fu et al.	OS and toxicity
Huang 2009 [28]	Patients with GC after curative surgery	TS, MTHFR	Asian (China)	NR	116	5-Fu+leucovorin et al.	OS
Ishida 2002 [29]	Patients with GC after surgery	TS	Asian (Japan)	NR	51	5-Fu et al.	OS
Keam 2008 [30]	Patients with recurrent or metastatic GC	GSTs, ERCC1-118	Asian (Korea)	WHO	73	5-Fu+leucovorin +oxaliplatin	RR and OS
Goekkurt 2009, [31]	Patients with advanced GC	TS, GSTs, MTHFR, ERCC1-118	European (Germany)	Others	134	5-Fu+leucovorin +oxaliplatin/cisplatin	RR, OS and toxicity
Ott 2006 [32]	Neoadjuvant chemotherapy for patients with locally advanced GC	TS, MTHFR	European (Germany)	Others	135	5-Fu+cisplatin	RR and OS
Ott 2011 [33]	Neoadjuvant chemotherapy for patients with locally advanced GC	MTHFR	European (Germany)	Others	144	5-Fu+leucovorin + cisplatin	OS
Lee 2005 [34]	Patients with advanced GC	TS, MTHFR	Asian (Korea)	NR	25	5-Fu et al.	OS

GC: gastric cancer; Others: evaluation criteria which were described in original papers; NR: not reporting; RR: response rate; OS: overall survival.

### Association between TS, MTHFR polymorphism and 5-Fu based chemotherapy

#### Response rate (RR)

Five studies (534 patients) reported the association between TS polymorphism and RR [11,14,19,31,32]. *P* value of heterogeneity test was 0.471 and a fixed-effect model was used. The pooled analysis showed that there was no significant difference between RR of patients with the 3R/3R genotype and that of patients with the 2R/3R and 2R/2R genotypes [(2R2R+2R3R)/3R3R: OR=0.92, 95% CI: 0.62–1.37]. Considering that RR might be influenced by ethnicity, evaluation criteria and the purpose of chemotherapy, we performed subgroup analysis. And no association was observed between TS polymorphism and RR by using the method of subgroup analysis (Table 2).

**Table 2:** **The association between TSpolymorphism and clinical outcomes**

**Study (reference)**	**RR (n/N)**	**OS (HR, 95% CI)**	**Toxicity**
Goekkurt 2006 [11]	3R3R: 3/12; 2R2R+2R3R: 9/32	NR	NR
Ruzzo 2006 [14]	3R3R: 22/61; 2R2R+2R3R: 48/114	NR	NR
Han 2010 [19]	3R3R: 16/28; 2R2R+2R3R: 5/10	NR	NR
Seo 2009 [21]	NR	NR	NSS
Shitara 2010 [27]	NR	(2R2R+2R3R)/3R3R: 1.28 (0.85, 1.96)	NSS
Huang 2009 [28]	NR	(2R2R+2R3R)/3R3R: 1.54 (0.879, 2.698)	NR
Ishida 2002 [29]	NR	(2R2R+2R3R)/3R3R: 1.26 (0.81, 1.95)	NR
Goekkurt 2009 [31]	3R3R: 17/33; 2R2R+2R3R: 35/101	NR	Grade 3/4 leukopenia: *P*=0.047
Ott 2006 [32]	3R3R: 10/41; 2R2R+2R3R: 23/94	2R2R/3R3R: 0.33 (0.22, 0.51); 2R3R/3R3R: 0.52 (0.37, 0.74)	NR
Lee 2005 [34]	NR	(2R2R+2R3R)/3R3R: 1.16 (0.68, 1.99)	NR
Combined analysis(OR/HR, 95CI %)	OR: (2R2R+2R3R)/3R3R	(2R2R+2R3R)/3R3R	___
	Total: 0.92 (0.62, 1.37);	Total: 1.29 (1.02, 1.64)	
	RECIST subgroup: 0.93 (0.33, 2.63);	All studies reported the data were Asian	
	Others subgroup: 0.92 (0.60, 1.41);	Palliative subgroup: 1.16 (0.68, 1.98)	
	Asian subgroup: 0.75 (0.18, 3.19);	Adjuvant subgroup: 1.33 (1.02, 1.73)	
	European subgroup: 0.94 (0.62, 1.41)		
	Palliative subgroup: 0.90 (0.58, 1.40)		
	Neoadjuvant subgroup: 1.00 (0.43, 2.36)		

RR: response rate; OS: overall survival; OR: odds ratio; HR: hazard ratio; NR: not reporting; NSS: no statistical significance.

Data of 5 included studies (571 patients) were applicable for analyzing the association between MTHFR polymorphism and RR [11,14,25,31,32]. *P* value of heterogeneity test was less than 0.1 and a random-effect model was used. Combined analysis demonstrated that there was no significant difference between RR of patients with C/C genotype and that of patients with the C/T and T/T genotype [(CT+TT)/CC: OR=1.12, 95% CI: 0.49–2.55]. In order to explore sources of heterogeneity, we performed subgroup analysis. The results of subgroup analysis showed that no significant association except Asian and WHO subgroups, where only one included studies reported [25] a significantly higher RR in C/T or T/T genotypes compared with the C/C genotype (OR=7.1, 95% CI: 1.5–33.53; Table 3).

**Table 3:** **The association between MTHFRpolymorphism and clinical outcomes**

**Study (reference)**	**RR(n/N)**	**OS (HR, 95% CI)**	**Toxicity**
Goekkurt 2006 [11]	CC: 10/28; CT+TT: 3/22	NR	NR
Ruzzo 2006 [14]	CC: 13/34; CT+TT: 57/141	NR	NR
Lu 2004 [25]	CC: 2/24; CT+TT: 20/51	NR	Nausea/vomiting: *P*=0.002
Shitara 2010 [27]	NR	TT/(CT+CC): 0.57 (0.33, 0.97)	NSS
Huang 2009 [28]	NR	TT/(CT+CC): 0.595 (0.349, 1.012)	NR
Goekkurt 2009 [31]	CC: 18/59; CT+TT: 34/75	NR	NSS
Ott 2006 [32]	CC: 16/50; CT+TT: 17/85	CT/CC: 1.8 (1.13, 2.88); TT/CC: 0.93 (0.54, 1.62)	NR
Ott 2011 [33]	NR	CT/CC: 0.8 (0.50, 1.36); TT/CC: 0.5 (0.18, 1.49)	NR
Lee 2005 [34]	NR	CT/CC: 0.91 (0.58, 1.43); TT/CC: 1.16 (0.65, 2.08)	NR
Combined analysis (OR/HR, 95CI %)	OR: (CT+TT)/CC	CT/CC (total): 1.10 (0.67, 1.79);	___
	Total: 1.12 (0.49, 2.55); RECIST subgroup: 0.28 (0.07, 1.20);	CT/CC (Asian subgroup): 0.91 (0.58, 1.43);	
	WHO subgroup: 7.1 (1.5, 33.53);	CT/CC (European subgroup): 1.21 (0.54, 2.67);	
	Others subgroup: 1.05 (0.51, 2.16);	CT/CC (Palliative subgroup): 0.91 (0.58, 1.43);	
	Asian subgroup: 7.1 (1.5, 33.53);	CT/CC (Neoadjuvant subgroup): 1.21 (0.54, 2.67) TT/CC (total): 0.94 (0.65, 1.37);	
	European subgroup: 0.85 (0.41, 1.77)	TT/CC (Asian subgroup): 1.16 (0.65, 2.08);	
	Palliative subgroup: 1.40 (0.55, 3.60);	TT/CC (European subgroup): 0.81 (0.50, 1.33);	
	Neoadjuvant subgroup: 1.12 (0.49, 2.55)	TT/CC (Palliative subgroup): 1.16 (0.65, 2.08);	
		TT/CC (Neoadjuvant subgroup): 0.81 (0.50, 1.33)	

RR: response rate; OS: overall survival; OR: odds ratio; HR: hazard ratio; NR: not reporting; NSS: no statistical significance.

#### Overall survival (OS)

Five studies (459 patients) reported the association between TS polymorphism and OS [27-29,32,34], but the data reported by Ott et al. could not be used for combined analysis [32], which showed that 2R/2R or 2R/3R genotypes were significantly associated with a favorable OS (2R2R/3R3R: HR=0.33, 95% CI: 0.22–0.51; 2R3R/3R3R: HR=0.52, 95% CI: 0.37–0.74). Therefore, data from 4 Asian studies (324 patients) were combined [27-29,34]. *P* value of heterogeneity test was 0.909 and a fixed-effect model was used. Meta-analysis showed that a significantly longer OS was observed in 3R/3R genotype compared with the 2R/2R or 2R/3R genotypes [(2R2R+2R3R)/3R3R: HR=1.29, 95% CI: 1.02–1.64; Table 2. (Additional file 1: Figure S1). When considering the purpose of chemotherapy, we found significant association in adjuvant subgroup but no significance in palliative subgroup (Table 2).

Data of 5 included studies (552 patients) were applicable for analyzing the association between MTHFR polymorphism and OS [27,28,32-34], but the data reported by Shitara et al. and Huang et al. could not be used for combined analysis [27,28]. These two studies reported patients with TT genotype had a longer OS compared with the C/T or C/C genotypes; although the difference was not statistically significant in the study by Huang et al. (Table 3). Therefore, data of 3 included studies (323 patients) were pooled [32-34]. Meta-analysis showed no significant association was seen between MTHFR polymorphism and OS (CT/CC: HR=1.10, 95% CI: 0.67–1.79; TT/CC: HR=0.94, 95% CI: 0.65–1.37). Additionally, subgroup analysis did not demonstrate significant difference (Table 3). (Additional file 2: Figure S2)

#### Toxicity

Because different evaluation criteria were used and very few studies reported the results, we did not combine the data. Three studies (360 patients) reported the association between TS polymorphism and toxicity [21,27,31]. Two studies found no significant genetic type was observed in conjunction with TS polymorphism [21,27]; but Goekkurt et al. reported that carriers of at least one 3R haplotype were at lower risk for developing grade 3/4 leukopenia with an OR of 0.12 (95% CI: 0.02-0.88) [31].

Data of 3 included studies (341 patients) were applicable for analyzing the association between MTHFR polymorphism and toxicity [25,27,31]. Two studies found no significant association between MTHFR polymorphism and toxicity [27,31]; however, Lu et al. reported that MTHFR TT was associated with higher frequency of nonhematologic toxicity (nausea/vomiting) [25].

#### Association between ERCC1, GSTs polymorphism and platinum based chemotherapy

##### Response rate (RR)

Seven studies (674patients) evaluated the association between ERCC1 polymorphism and RR [11,14,18,19,21,30,31]. All of the patients underwent palliative chemotherapy. *P* value of heterogeneity test was 0.696 and a fixed-effect model was used. The pooled OR for RR was 0.77 (95% CI: 0.54–1.11 Table 4), which suggested that there was no significant association between ERCC1 polymorphism and RR. Subgroup analysis was performed according to ethnicity and evaluation criteria. The pooled OR was 0.56 (95% CI: 0.32–0.97) for European subgroup and 0.56 (95% CI: 0.32–1.00) for “Others” subgroup, which suggested that RR was significantly higher in C/C genotype compared with C/T or T/T genotypes. However, the difference was not statistically significant in Asian, RECIST or WHO subgroup (Table 4).

**Table 4:** **The association between ERCC1polymorphism and clinical outcomes**

**Study (reference)**	**RR (n/N)**	**OS (HR, 95% CI)**	**Toxicity**
Goekkurt 2006 [11]	CC: 2/5; CT+TT: 11/44	NR	NR
Ruzzo 2006 [14]	CC: 21/38; CT+TT: 49/137	NR	NR
Park 2011 [18]	CC: 35/64; CT+TT: 23/44	TC/CC: 0.94 (0.556, 1.587); TT/CC: 1.918 (0.748, 4.919)	NR
Han 2010 [19]	CC: 12/23; CT+TT: 9/15	NR	NR
Stocker 2009 [20]	NR	CT/CC: 0.72 (0.40, 1.31); TT/CC: 1.07 (0.59, 1.95)	NR
Seo 2009 [21]	CC: 11/42; CT+TT: 7/33	NR	NSS
Liu 2011 [22]	NR	(CT+TT)/CC: 2.388 (1.448, 3.937)	NR
Huang 2009 [26]	NR	(CT+TT)/CC: 1.072 (0.620, 1.855)	NR
Keam 2008 [30]	CC: 17/40; CT+TT: 15/33	(CT+TT)/CC: 1.251 (0.68, 2.302)	NR
Goekkurt 2009 [31]	CC: 9/21; CT+TT: 43/113	NR	NSS
Combined analysis (OR/HR, 95CI %)	OR: (CT+TT)/CC	HR: (CT+TT)/CC	___
	Total (Palliative chemotherapy): 0.77 (0.54, 1.11);	Total: 1.5 (0.90, 2.49)	
		Palliative subgroup: 1.77 (0.94, 3.33);	
	RECIST subgroup: 0.89 (0.52, 1.53);	Adjuvant subgroup: 1.07 (0.62, 1.85);	
	WHO subgroup: 1.13 (0.45, 2.85);		
		All studies reported the data were	
	Others subgroup: 0.56 (0.32, 1.00);	Asian.	
	Asian subgroup: 0.98 (0.61, 1.59);		
	European subgroup: 0.56 (0.32, 0.97)		

RR: response rate; OS: overall survival; OR: odds ratio; HR: hazard ratio; NR: not reporting; NSS: no statistical significance.

Six studies (794 patients) evaluated the association between GSTM1 polymorphism and RR [11,14,17,21,23,31]. *P* value of heterogeneity test was 0.734 and a fixed-effect model was used. The pooled OR for RR was 1.16 (95% CI: 0.85–1.58; Table 5), which suggested that there was no significant association between RR of patients with M- genotype and that of patients with the M+ genotype. Subgroup analysis according to ethnicity, evaluation criteria and chemotherapy purpose also did not show significant association (Table 5).

**Table 5:** **The association between GSTspolymorphisms and clinical outcomes**

**Study (reference)**	**RR (n/N)**	**OS (HR, 95%CI)**	**Toxicity**
Goekkurt 2006 [11]	GSTM1: M-: 9/32; M+: 4/18	NR	NR
	GSTP1: AA: 7/30; GA+GG: 6/18	GG/(GA+AA): 0.65 (0.43, 1.00)	NR
	GSTT1: T-: 8/38; T+: 5/12	NR	NR
Ruzzo 2006 [14]	GSTM1: M-: 36/78; M+: 34/97	NR	NR
	GSTP1: AA: 20/87; GA+GG: 50/88	GG/AA: 0.58 (0.43, 0.80)	NR
	GSTT1: T-: 6/21; T+: 64/154	GA/AA: 0.54 (0.40, 0.74)	NR
		NR	
Shim 2010 [17]	GSTM1: M-: 48/124; M+: 29/76	M-/M+: 1.10 (0.80, 1.51)	NR
	GSTP1: AA: 46/133	AG/AA: 1.12 (0.79, 1.58)	NR
	GA+GG: 31/67	GG/AA: 0.76 (0.33, 1.77)	NR
	GSTT1: T-: 40/106; T+: 37/94	T-/T+: 0.77 (0.57, 1.06)	
Seo 2009 [21]	GSTM1: M-: 12/49; M+: 6/26	NR	NR
	GSTP1: AA: 10/47; GA+GG: 8/28	NR	NR
	GSTT1: T-: 8/39; T+: 10/36	NR	NR
Liu 2011 [22]	GSTP1: NR	(GG+AG)/AA: 0.53 (0.36, 0.80)	NR
Ott 2008 [23]	GSTM1: M-: 15/52; M+: 13/60	M-/M+: 1.38 (0.92, 2.08)	NR
	GSTP1: AA: 12/55; GA+GG: 21/77	AG/AA: 0.80 (0.55, 1.15)	NR
	GSTT1: T-: 5/23; T+: 24/87	GG/AA: 0.95 (0.53, 1.71)	NR
		T-/T+: 1.09 (0.69, 1.72)	
Li 2010 [24]	GSTP1: AA: 17/44; GA+GG: 29/41	(GG+AG)/AA: 0.44 (0.25, 0.78)	SS
Huang 2009 [26]	GSTM1: NR	M-/M+: 1.425 (0.822, 2.469)	NR
	GSTP1: NR	(GG+AG)/AA: 0.471 (0.252, 0.878)	NR
Keam 2008 [30]	GSTP1: AA: 22/44; GA+GG: 10/29	(GG+AG)/AA: 0.621 (0.452, 1.606)	NR
Goekkurt 2009 [31]	GSTM1: M-: 26/72; M+: 26/62	NR	NR
	GSTP1: AA: 26/64; GA+GG: 26/69	NR	SS
	GSTT1: T-: 5/23; T+: 47/111	T-/T+: 1.94 (1.14, 3.32)	NR
Combined analysis(OR/HR, 95CI%)	**GSTM1(OR), M-/M+** Total: 1.16 (0.85, 1.58)	**GSTM1(HR), M-/M+** Total: 1.23 (0.98, 1.55)	___
	RECIST subgroup: 1.07 (0.66, 1.74)	Asian subgroup: 1.17 (0.89, 1.55)	
	Others subgroup: 1.23 (0.82, 1.84)	European subgroup: 1.38 (0.92, 2.07)	
	Asian subgroup: 1.04 (0.62, 1.74)	Palliative subgroup: 1.10 (0.80, 1.51)	
	European subgroup: 1.24 (0.84, 1.82)	Adjuvant subgroup: 1.42 (0.82, 1.47)	
	Palliative subgroup: 1.04 (0.73, 1.49)	Neoadjuvant subgroup: 1.38 (0.92, 2.07)	
	Neoadjuvant subgroup: 1.59 (0.86, 2.92)	**GSTP1(HR), (GG+AG)/AA,** Total: 0.51 (0.39, 0.67)	
	**GSTP1(OR), (GG+AG)/AA**		
		Palliative subgroup: 0.52 (0.39, 0.70)	
		Adjuvant subgroup: 0.47 (0.25, 0.88)	
	Total: 1.63 (0.98, 2.70)	All studies reported the data were Asian.	
	RECIST subgroup: 1.60 (0.98, 2.60)	**GSTP1(HR), GG/AA,** Total: 0.66 (0.51, 0.85)	
	WHO subgroup: 0.53 (0.20, 1.38)		
	Others subgroup: 2.1 (0.93, 4.74)	Asian subgroup: 0.76 (0.33, 1.76)	
	Asian subgroup: 1.51 (0.72, 3.16)	European subgroup: 0.65 (0.49, 0.85)	
	European subgroup: 1.74 (0.77, 3.91)	Palliative subgroup: 0.60 (0.45, 0.80)	
	Palliative subgroup: 1.67 (0.93, 2.99)	Neoadjuvant subgroup: 0.95 (0.53, 1.71)	
	Neoadjuvant subgroup: 1.34 (0.60, 3.03)	**GSTP1(HR), AG/AA,** Total: 0.78 (0.51, 1.20)	
	**GSTT1(OR), T-/T+** Total: 0.67 (0.47, 0.97)		
	RECIST subgroup: 0.79 (0.49, 1.27)	Asian subgroup: 1.12 (0.79, 1.58)	
	Others subgroup: 0.53 (0.29, 0.97)	European subgroup: 0.65 (0.44, 0.95)	
	Asian subgroup: 0.87 (0.52, 1.43)	Palliative subgroup: 0.77 (0.38, 1.58)	
	European subgroup: 0.51 (0.29, 0.88)	Neoadjuvant subgroup: 0.80 (0.55, 1.16)	
	Palliative subgroup: 0.67 (0.45, 0.99)	**GSTT1(HR), T-/T+** Total: 1.14 (0.68, 1.90)	
	Neoadjuvant subgroup: 0.73 (0.24, 2.18)		
		Asian subgroup: 0.77 (0.56, 1.05)	
		European subgroup: 1.43 (0.81, 2.51)	
		Palliative subgroup: 1.19 (0.48, 2.94)	
	Neoadjuvant subgroup: 1.09 (0.69, 1.72)		

RR: response rate; OS: overall survival; OR: odds ratio; HR: hazard ratio; NR: not reporting; SS: statistical significance.

Data of 8 studies (959 patients) could be used for evaluating the association between GSTP1 polymorphism and RR [11,14,17,21,23,24,30,31]. *P* value of heterogeneity test was less than 0.1 and a random-effect model was used. The pooled OR for RR was 1.63 (95% CI: 0.98–2.70; Table 5), which suggested that patients with G/G or A/G genotype had a higher RR compared with A/A genotype, although the difference was not statistically significant. The result was not changed by using the method of subgroup analysis according to ethnicity, evaluation criteria and chemotherapy purpose (Table 5).

Data of 6 studies (794 patients) were available for GSTT1 [11,14,17,21,23,31]. *P* value of heterogeneity test was 0.664 and a fixed-effect model was used. Combined analysis suggested that RR was higher in T+ genotype compared with T- genotype (T-/T+: OR=0.67, 95% CI: 0.47–0.97; Table 5). When used the method of subgroup analysis, the result was changed in Asian, RECIST and neo-adjuvant subgroups, but not changed in European or “Others” subgroups (Table 5).

### Overall survival

Five studies (587 patients) evaluated the association between ERCC1 polymorphism and OS [18,20,22,26,30], but the studies by Park et al. and Stocker et al. could not be used for meta-analysis, which reported no significant association was observed between ERCC1 polymorphism and OS [18,20]. Therefore 3 Asian studies (301 patients) were used for combined analysis [22,26,30]. *P* value of heterogeneity test was less than 0.1 and a random-effect model was used. Meta-analysis showed that patients with C/C genotype had a longer OS compared with C/T or T/T genotypes; however, the difference was not statistically significant [(CT+TT)/CC: HR=1.50, 95% CI: 0.90–2.49; Table 4. The results were not changed by subgroup analysis considering chemotherapy purpose. Sensitivity analysis identified that the study reported by Liu et al. [22] was the main source of heterogeneity. (Additional file 3: Figure S3)

Data of 3 studies (441 patients) could be used for assessing the association between GSTM1 polymorphism and OS [17,23,26]. *P* value of heterogeneity test was 0.59 and a fixed-effect model was used. Meta-analysis showed a longer OS in M+ genotype compared with M- genotype; however, the difference was not statistically significant (M-/M+: HR=1.23, 95% CI: 0.98–1.55; Table 5). The result was consistent by using the method of subgroup analysis considering ethnicity and chemotherapy purpose (Table 5). (Additional file 4: Figure S4)

Eight studies (959 patients) reported evaluated the association between GSTP1 polymorphism and OS [11,14,17,22-24,26,30]. Among them 4 studies (393 patients) used dominant model (GG/AG vs AA) [22,24,26,30], three (514 patients) used codominant model (GG vs AA, AG vs AA) [14,17,23], and one (52 patients) used recessive model [11]. Therefore, we combined the data of included studies which used dominant and codominant model respectively. The results of meta-analysis showed that G/G or G/A genotypes were associated with a longer OS compared with A/A genotype (Table 5). (Additional file 5: Figure S5)

HR of 3 studies (473 patients) were available for GSTT1 [17,23,31]. *P* value of heterogeneity test was less than 0.1 and a random-effect model was used. Combined analysis suggested that there was no significant association between GSTT1 polymorphism and OS [T-/T+: HR=1.14, 95% CI: 0.68–1.90; Table 5. However, in Asian subgroup we could see a longer OS without statistical significance in T- genotype compared with T+ genotype (Table 5). (Additional file 6: Figure S6)

### Toxicity

Data of 2 included studies (228 patients) were applicable for analyzing the association between ERCC1-118 polymorphism and toxicity [21,31], and no significant association was identified. Two studies (226 patients) evaluated the association between GSTP1 polymorphism and toxicity [24,31], and they both found that patients with GSTP1-105 A/A genotype were at significantly higher risk of experiencing hematological and neurological toxicity compared with patients with A/G or G/G genotype.

## Discussion

Recently, a growing body of evidence suggests interindividual variation in drug-metabolizing enzymes (such as TS and MTHFR), nucleotide excision repair systems (such as ERCC1), and GST families may affect anticancer drug efficacy for GC. However, the association remains controversial and uncertain. Therefore, we conducted this systematic review aiming to provide a comprehensive and up-to-date overview on the biomarkers that can be served as predictive surrogates for clinical outcomes in patients with GC. To our knowledge, this is the first meta-analysis evaluating genetic polymorphisms in predicting clinical outcomes of GC patients treated with platinum/5-Fu-based chemotherapy. In this study, we found TS and MTHFR polymorphisms were associated with clinical outcomes of 5-Fu based chemotherapy, and sequences for ERCC1 and GSTs had a relationship with clinical outcomes of platinum based chemotherapy.

The strengths of this systematic review were its well defined search strategy and selection of study according to the strict inclusion criteria. In addition, we performed subgroup analysis in order to reduce heterogeneity caused by ethnicity and evaluation criteria. These factors increased the reliability of our review. However, our study was not faultless. Firstly, potential selection bias was introduced because of different inclusion criteria in included studies. For example, Stocker et al. [20] included locally advanced GC patients without distant metastasis, but Shim et al. [17] included patients with recurrent or metastatic GC. Secondly, chemotherapy regimen was different among included studies, but this factor was not taken into consideration when performing meta-analysis in lack of coherence of primary data. Finally, it was impossible for us to identify all relevant literatures, even though we made great efforts. Moreover, publication bias might exist and we did not draw a funnel plot because of uncomplete data in included studies.

The fluoropyrimidine 5-Fu has been the standard agent in GC chemotherapy, either as a single drug or in combination with other agents. TS, the rate-limiting enzyme in de novo pyrimidine biosynthesis, is a target enzyme of 5-FU. Recent evidence indicates that elevated TS in GC, in both mRNA and protein levels, are associated with clinical resistance to 5-Fu and consequently with poor outcome of the patients receiving 5-FU therapy [29]. And high TS expression is now well known to be associated with polymorphism of the 28-base pair tandem repeat sequence (VNTR) in the TS promoter enhancer region (TSER) [35]. The presence of triple repeats (3R/3R) has been shown to be associated with higher TS expression [35], with resultant lower fluorouracil efficacy [36], while double repeat homozygous (2R/2R) has provided better clinical outcomes after 5-Fu-based chemotherapy [37]. In our studies, we found there was no significant association between TS polymorphism in VNTR and RR [(2R2R+2R3R)/3R3R: OR=0.92, 95% CI: 0.62–1.37]; and the result was not changed by using the method of subgroup analysis according to ethnicity, evaluation criteria or chemotherapy purpose. However, a significantly longer OS was observed in 3R/3R genotype compared with the 2R/2R or 2R/3R genotypes [(2R2R+2R3R)/3R3R: HR=1.29, 95% CI: 1.02–1.64]. This result was opposite to previous reports [37]. Recently, some researchers found similar phenomena with ours, considering survival or response to 5-Fu treatment in patients with 3R/3R gene polymorphism better than [38], or equal to that with the 2R/2R genotype [39]. This contrary phenomena suggests that the whole transcriptional activity of TS is not always dependent on the number of the tandem repeats alone. And some other patterns of polymorphism, including polymorphism of 6-base pair (bp) insertion (6+/6+ genotype) in the 3’ untranslated region and a G/C polymorphism in the 3R VNTR allele can possibly explain these inconclusive data [40]. Therefore, a single polymorphism of TS is not sufficient to explain changes in the clinical benefit of 5-Fu, and complex combinations of variants should be considered.

MTHFR is another central enzyme for maintaining DNA integrity and stability by regulating the folate pool. Studies found that MTHFR C677T single-nucleotide polymorphism (alanine to valine substitution at codon 222) was associated with reduced enzymatic activity [41]. Theoretically, decreased MTHFR activity confers a more effective TS inhibition and resultant increased 5-Fu efficacy. And this was confirmed by previous studies, which reported the highest clinical RR to 5-Fu in subjects with TT mutant homozygous [42]. However, our meta-analysis showed that there was no correlation between MTHFR C677T single-nucleotide polymorphism and clinical outcomes of 5-Fu-based chemotherapy, whether RR [(CT+TT)/CC: OR=1.12, 95% CI: 0.49–2.55] or OS (CT/CC: HR=1.10, 95% CI: 0.67–1.79; TT/CC: HR=0.94, 95% CI: 0.65–1.37). Considering chemotherapy purpose and the variation of allele frequency in different ethnicity (TT is 1% or less among Blacks from Africa or the United States, and 10% in Caucasians) [43], we conducted subgroup-analysis but failed to reveal significant association (Table 3). As was discussed for TS polymorphism, the discrepant results may be ascribed to other gene polymorphism regulating MTHFR activity, such as A1298C single-nucleotide polymorphism.

Platinum derivatives, mainly cisplatin but more recently oxaliplatin, have been widely used for treating GC. Resistance to platinum is attributable to enhanced DNA repair. Genes of the NER pathway plays a key role in recognition and repair of damaged DNA caused by platinum compounds. Functional polymorphism of ERCC1-C118T has been demonstrated to impact clinical outcome of patients receiving platinum-based chemotherapy [44]. Some researchers found that patients with the ERCC1 118 T/T genotype were more likely to respond to oxaliplatin-based chemotherapy than carriers of the other genotypes in colorectal and pancreatic cancer [45,46]. However, several studies indicated that no significant association was found between ERCC1 codon 118 polymorphism and platinum sensitivity [14,47]. In this study, we found no significant association between ERCC1 codon 118 polymorphism and clinical outcomes (Table 4). Nevertheless, a higher response rate was found in patients with C allele in European subgroup [(CT+TT)/CC: OR=0.56, 95% CI: 0.32–0.97]. This was consistent with a recent study on advanced GC treated with fluorouracil/cisplatin palliative chemotherapy [48]. The possible reasons for controversial results is that ERCC1 codon 118 polymorphism is in linkage disequilibrium with other ERCC1 mutations or polymorphisms that directly affect its expression can not be ruled out. Other possible reasons may be variable doses and schedules of platinum-based therapy, different kind of cancers and variable tumor stages. Studies with large sample size using the method of multi-variant analyses may help us to give more persuasive data on the putative association in future.

Through conjugation to glutathione, GST is a member of isozymes’ family which plays an important role in the detoxification of platinum-based chemotherapy. Some isoenzymes (in particular GSTM1, P1 and T1) are involved in this process. It has been reported that the GSTP1-A105G polymorphism was associated with prognosis of gastric and colorectal cancer patients receiving platinum-based chemotherapy (the mutant 105 G/G homozygous involving with survival benefits [11,49], while wild type 105 A/A homozygous associated with unfavorable clinical outcomes [14]). In this study, we found that G/G or G/A genotypes were associated with a longer OS compared with A/A genotype [(GG+AG)/AA: HR=0.51, 95% CI: 0.39–0.67], which was concordant with previous studies. With regard to polymorphism in the GSTT1 and GSTM1, which lead to complete loss of enzymatic activity, the results are divergent. Null genotypes of GSTM1 and GSTT1 could provide significant survival benefit in breast cancer [50], but other studies in colorectal cancer reported inconsistent results [49,51]. We found that there was no significant association between GSTM1 polymorphism and clinical outcomes; however, RR was higher in T+ genotype compared with T- genotype [T-/T+: OR=0.67, 95% CI: 0.47–0.97; Table 5. These discrepant results may be ascribed to differences in the distribution of the GST families and differences in enzymatic activity for drug detoxification in various tissues.

Multiple genes are involved in the mechanisms with complex interplay. Despite still being in the investigational stage, efforts for predicting clinical outcomes using expression profiles of multiple key genes have been also intensively performed in various malignancies, including GC [11,14,26,52]. This movement from single gene polymorphism to a more comprehensive pathway evaluation could undoubtedly offer a more tailored approach to chemotherapy by providing a more effective biomarker through a better understanding of the genetic and molecular basis underlying variable drug response among patients, and ultimately improve treatment outcomes. Meanwhile, we must notice that never single agents are delivered. Poly-chemotherapy which combines several drugs (mainly 5-Fu and platinum) is the main chemotherapy regime currently. Whether the effect of genetic polymorphisms will change because of drug interactions is worthy of studying. In addition, attentions should be paid for the association between genetic polymorphisms and adverse events. In this meta-analysis, very few included studies evaluated this association, and the evaluation criteria were different. Therefore, we just described the results of included studies without combined analysis. More studies using uniform evaluation standard are needed to assess the association between genetic polymorphisms and chemotherapy toxicity in future.

## Conclusion

In conclusion, polymorphisms of ERCC1, GSTs, TS and MTHFR were closely associated with clinical outcomes of GC patients treated with platinum/5-Fu-based chemotherapy. Studies with large sample size using the method of multi-variant analyses may help us to give more persuasive data on the putative association in future. Additionally, targeted agents may offer new tools in GC treatment under the circumstance of inevitable side effects of chemotherapy.

## Competing interests

The authors declare that they have no competing interests.

## Authors’ contributions

Zhen Wang, Jun-qiang Chen and Xin-gan Qin designed this study; Zhen Wang, Jun-qiang Chen and Xin-gan Qin performed this research; Zhen Wang and Jun-qiang Chen analyzed the data; Xin-gan Qin interpreted the results; Zhen Wang drafted the manuscript; Jin-lu Liu and Yuan Huang revised the paper. All the authors approved the final manuscript.

## Pre-publication history

The pre-publication history for this paper can be accessed here:

http://www.biomedcentral.com/1471-230X/12/137/prepub

## Supplementary Material

Additional file 1 **Figure S1. **The association between TS polymorphism and OS in patients receiving 5-Fu based chemotherapy [(2R2R+2R3R)/3R3R]. Click here for file

Additional file 2 **Figure S2. **The association between MTHFR polymorphism and OS in patients receiving 5-Fu based chemotherapy (a: CT/CC; b: TT/CC). Click here for file

Additional file 3 **Figure S3. **The association between ERCC1 polymorphism and OS in patients receiving platinum based chemotherapy [(CT+TT)/CC]. Click here for file

Additional file 4 **Figure S4.** The association between GSTM1 polymorphism and OS in patients receiving platinum based chemotherapy (M-/M+). Click here for file

Additional file 5 **Figure S5.** The association between GSTP1 polymorphism and OS in patients receiving platinum based chemotherapy [a: (GG+AG)/AA; b: GG/AA; c: AG/AA]. Click here for file

Additional file 6 **Figure S6.** The association between GSTT1 polymorphism and OS in patients receiving platinum based chemotherapy (T-/T+). Click here for file
