# Supplementary material for: Polymorphisms in ERCC1, GSTs, TS and MTHFR predict clinical outcomes of gastric cancer patients treated with platinum/5-Fu-based chemotherapy: a systematic review
Source: BMC Gastroenterol. 2012 Sep 29;12:137. doi: 10.1186/1471-230X-12-137 (PMC3524027; doi:10.1186/1471-230X-12-137)
Supplement: Additional file 2 — Figure S2. The association between MTHFR polymorphism and OS in patients receiving 5-Fu based chemotherapy (a: CT/CC; b: TT/CC). [file 1471-230X-12-137-S2.doc]

a

b
