# Supplementary material for: Polymorphisms in ERCC1, GSTs, TS and MTHFR predict clinical outcomes of gastric cancer patients treated with platinum/5-Fu-based chemotherapy: a systematic review
Source: BMC Gastroenterol. 2012 Sep 29;12:137. doi: 10.1186/1471-230X-12-137 (PMC3524027; doi:10.1186/1471-230X-12-137)
Supplement: Additional file 5 — Figure S5. The association between GSTP1 polymorphism and OS in patients receiving platinum based chemotherapy [a: (GG+AG)/AA; b: GG/AA; c: AG/AA]. [file 1471-230X-12-137-S5.doc]

a

b

c
