# Supplementary material for: Polymorphisms in ERCC1, GSTs, TS and MTHFR predict clinical outcomes of gastric cancer patients treated with platinum/5-Fu-based chemotherapy: a systematic review
Source: BMC Gastroenterol. 2012 Sep 29;12:137. doi: 10.1186/1471-230X-12-137 (PMC3524027; doi:10.1186/1471-230X-12-137)
Supplement: Supplementary file 3 [file 1471-230X-12-137-S1.doc]

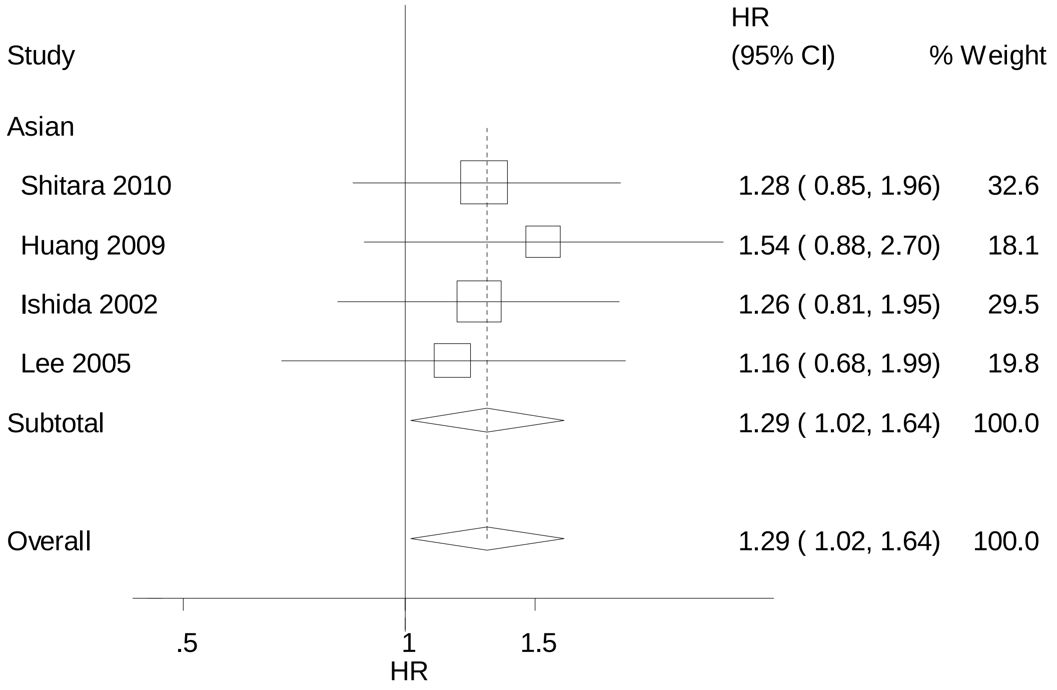
HR
(95% CI)
Study
% Weight
Asian
Shitara 2010
1.28 (0.85, 1.96)
32.6
Huang 2009
1.54 (0.88, 2.70)
18.1
Ishida 2002
1.26 (0.81, 1.95)
29.5
Lee 2005
1.16 (0.68, 1.99)
19.8
Subtotal
1.29 (1.02, 1.64)
100.0
Overall
1.29 (1.02, 1.64)
100.0
.5
1
1.5
HR
