# Supplementary material for: Polymorphisms in ERCC1, GSTs, TS and MTHFR predict clinical outcomes of gastric cancer patients treated with platinum/5-Fu-based chemotherapy: a systematic review
Source: BMC Gastroenterol. 2012 Sep 29;12:137. doi: 10.1186/1471-230X-12-137 (PMC3524027; doi:10.1186/1471-230X-12-137)
Supplement: Supplementary file 4 [file 1471-230X-12-137-S3.doc]

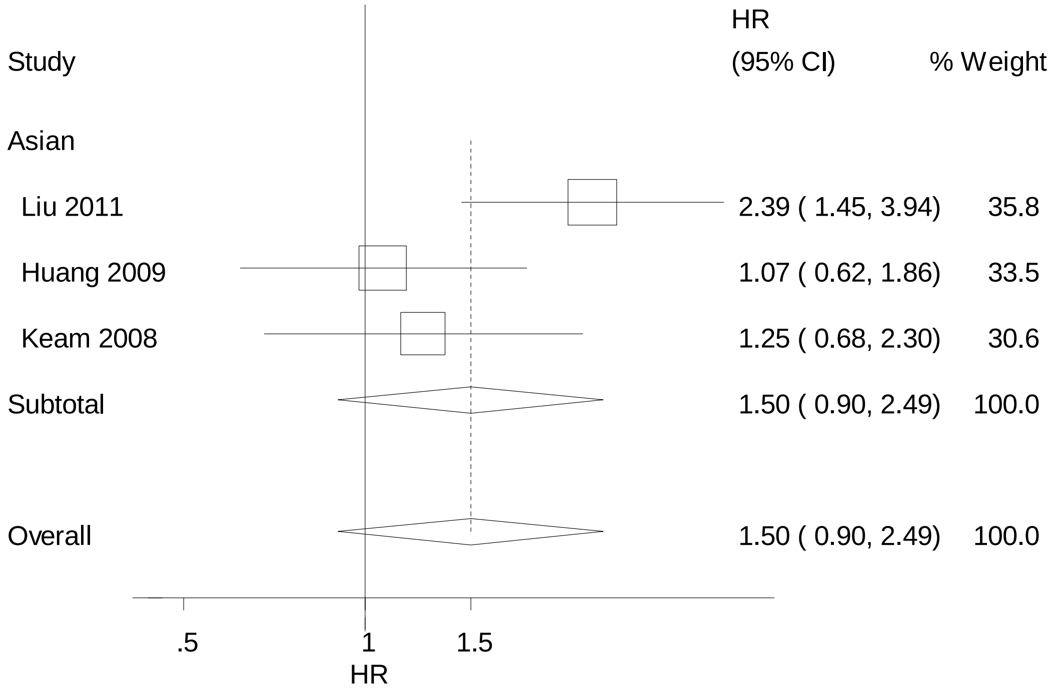

HR
Study
(95% CI)
% Weight
Asian
Liu 2011
2.39 (1.45, 3.94)
35.8
Huang 2009
1.07 (0.62, 1.86)
33.5
Keam 2008
1.25 (0.68, 2.30)
30.6
Subtotal
1.50 (0.90, 2.49)
100.0
Overall
1.50 (0.90, 2.49)
100.0
.5
1
1.5
HR
