# Supplementary material for: Polymorphisms in ERCC1, GSTs, TS and MTHFR predict clinical outcomes of gastric cancer patients treated with platinum/5-Fu-based chemotherapy: a systematic review
Source: BMC Gastroenterol. 2012 Sep 29;12:137. doi: 10.1186/1471-230X-12-137 (PMC3524027; doi:10.1186/1471-230X-12-137)
Supplement: Supplementary file 6 [file 1471-230X-12-137-S6.doc]

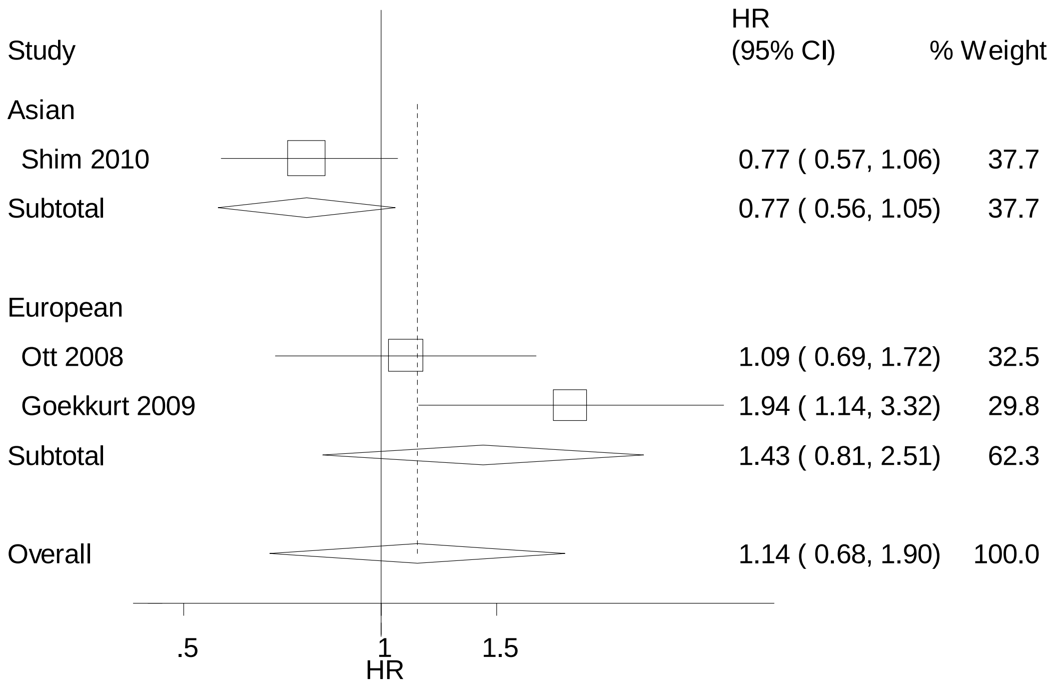
Study
HR
(95% CI)
% Weight
Asian
Shim 2010
0.77 (0.57, 1.06)
37.7
Subtotal
0.77 (0.56, 1.05)
37.7
European
Ott 2008
1.09 (0.69, 1.72)
32.5
Goekkurt 2009
1.94 (1.14, 3.32)
29.8
Subtotal
1.43 (0.81, 2.51)
62.3
Overall
1.14 (0.68, 1.90)
100.0
.5
1
1.5
HR
